# A Novel Approach to Diagnostic and Operative Hysteroscopy Training: Hands-On Training With One-to-One Tutoring in Live Patients

**DOI:** 10.7759/cureus.88205

**Published:** 2025-07-17

**Authors:** Spyridon Kavrochorianos, Olga Triantafyllidou, Stamatia Chasiakou, Evangelia Panagodimou, Panagiotis Christopoulos, Theodoros Kalampokas, Emmanouil Kalampokas, Dimitrios Balafoutas, Grigorios Karampas, Nikolaos Vlahos

**Affiliations:** 1 Third Department of Obstetrics and Gynecology, Elena Venizelou General and Maternity Hospital, Athens, GRC; 2 Second Department of Obstetrics and Gynecology, Aretaieion University Hospital, National and Kapodistrian University of Athens, Athens, GRC; 3 Department of Family Medicine, Hippocratio General Hospital, Athens, GRC

**Keywords:** gynecological procedures, hands-on experience, hysteroscopy, training, uterine pathology

## Abstract

Introduction: Hysteroscopy is the gold standard technique for the evaluation and treatment of intrauterine pathology related to infertility, abnormal bleeding, and recurrent miscarriages. Hysteroscopy training is a fundamental part of training in gynecology. We report an innovative live-patient hysteroscopy training curriculum developed by the Second Department of Obstetrics and Gynecology of ''Aretaieion'' University Hospital of Athens, Greece.

Methods: The program includes a two-day course integrating both theory and hands-on sessions. The theoretical component encompasses pelvic anatomy, instrument-related physics, recognition of uterine pathology, indications of hysteroscopy, patient preparation, use of ultrasound, management of complications, and post-surgery care, promoting a deeper comprehension of limitations and applications of hysteroscopy. On the first day of practical training, trainees use hysteroscopic instruments in small groups in a dry lab under the supervision of experienced tutors. On the second day, they move on to live-patient cases for diagnostic and surgical hysteroscopy. Participants are exposed to a wide spectrum of surgical interventions ranging from simple diagnostic hysteroscopy to more advanced procedures such as International Federation of Gynecology and Obstetrics (FIGO) type 2 myomectomy or intrauterine adhesion management.

Results: Post-course evaluation via a questionnaire has served as a valuable aid in the continuous improvement of the training program, in line with the ever-evolving field of hysteroscopic surgery. The overall satisfaction of the participants according to the self-reported 5-point verbal numeric rating scale (VNRS-5) was very high (mean score 4.8).

Conclusion: Our program addresses the lack of sufficient hysteroscopy training and offers an alternative to simulation-based training. Its curriculum is pioneering, combining theoretical as well as hands-on training with one-to-one tutoring in live patients, facilitating gynecologists to incorporate hysteroscopic procedures with more confidence into their surgical practice.

## Introduction

Hysteroscopy is a minimally invasive procedure that allows direct visualization of the uterine cavity. It is an invaluable aid in the accurate diagnosis and treatment of various uterine pathologies, such as endometrial polyps, submucosal fibroids, intrauterine adhesions, and congenital uterine anomalies, resulting in infertility, abnormal bleeding, or recurrent miscarriages [1-7]. Due to its safety and efficacy, hysteroscopy is considered the gold standard for evaluating and managing intrauterine pathology in everyday gynecological practice.

Exposure to proper training and mastering new techniques are essential to young gynecologists and even to experienced practitioners. Compared to most gynecological operative procedures, hysteroscopy has a minimal risk of complications [8]. This renders advanced operative procedures, such as hysteroscopic adhesiolysis and myomectomies, relatively safe [9,10]. On the other hand, hysteroscopic procedures may result in serious complications related to suboptimal surgical skills [11].

Training in hysteroscopy is essential to ensure patient safety and proficient practice in this specialized field [12]. Proficiency in hysteroscopy, however, demands comprehensive training. That includes acquiring practical skills and gaining hands-on experience. Therefore, specialized training programs are essential.

Despite advances in medical education, a significant proportion of Obstetrics and Gynecology graduates (approximately 28.2%) still report a lack of confidence in their surgical skills post-residency, especially in hysteroscopy [13]. One of the primary obstacles in hysteroscopy training is the lack of a universally recognized and validated curriculum for hysteroscopy training. While various assessment tools and methods have been developed, there is still a need for a standardized approach [12,14]. Hysteroscopy training presents unique challenges, requiring surgeons to achieve proficiency in operating within a two-dimensional space, using a fixed access point, and applying a limited range of movements. Therefore, specialized skills and excellent hand-eye coordination are required to ensure efficacy and patient safety [15]. To address these challenges, various models of training in hysteroscopy have been developed [16-18]. However, most of these courses utilize primarily virtual reality (VR) simulators or dry labs, offering limited hands-on experience in real-life surgical settings. Some training models attempt to simulate the hysteroscopic environment using animal organs, fruits and vegetables, or synthetic uteri [17]. Although these methods provide some level of practical experience, they often fall short in imparting the specific skills and in-depth knowledge required for proficient hysteroscopic surgery. As a result, there is a growing need for more comprehensive and realistic training programs that not only equip gynecologists with the necessary technical skills but also build their confidence to apply these skills effectively and safely in real-life settings.

In the present study, we demonstrated a novel curriculum on hysteroscopy training, aiming to complement gynecologists’ skills in hysteroscopy. The pedagogical framework underlying this curriculum involves the acquisition of theoretical knowledge in hysteroscopy as well as practical hands-on experience in training simulators and one-to-one tutoring on live patients. This program is tailored for young gynecologists who have recently finished their residency and wish to improve their skills in hysteroscopy. Also, this course could be useful for experienced doctors who want to advance their surgical skills in more complex procedures such as myomectomy of International Federation of Gynecology and Obstetrics (FIGO) type 2 myomas or management of Asherman’s syndrome.

The primary objective of this study was to describe and evaluate a novel, structured curriculum for hysteroscopy training incorporating theoretical education, simulation-based practice, and one-to-one supervised live-patient procedures. Secondary aims included addressing the lack of confidence among new gynecologists in performing hysteroscopy [13] and providing an effective alternative to simulation-only models that may not fully prepare trainees for real-life surgical challenges.

## Materials and methods

Our training program is a two-day course conducted by the Second Department of Obstetrics and Gynecology of “Aretaieion” University Hospital of Athens, Greece, in collaboration with local private hospitals. The course abides by the recommendations of the Gynaecological Endoscopic Surgical Education and Assessment (GESEA) and is conducted in full compliance with the ethical standards outlined in the Declaration of Helsinki. The study protocol was reviewed and approved by the Hospital Ethics Committee. All patients involved in the training program are thoroughly informed about the course objectives, and each of them provides written informed consent prior to participation.

The inclusion criteria of the practical part (second day) of our course were based strictly on predefined inclusion criteria rather than random sampling. Only gynecologists who had completed their residency and provided proof of insurance coverage were eligible for the hands-on portion of the training. Clinical practitioners of other specialties, midwives, or residents are allowed to participate only in the theoretical part and the hands-on session in training simulators during the first day of the course.

**Figure 1:**
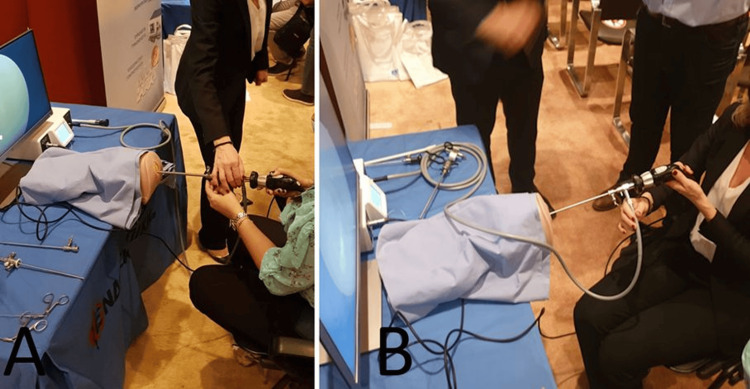
(A, B) Hands-on session on a surgical model, with live video presentation. In this keyhole case simulation, camera navigation allows better hand-eye coordination to develop hysteroscopic psychomotor skills.

During the first day, the theoretical part takes place with short and informative lectures including various principles and topics in hysteroscopy (Table 1). After the theoretical session and discussion between trainees and trainers, video-based observational modules featuring real-case hysteroscopies with step-by-step explanations were analyzed and discussed with emphasis on the insertion of a 30° optics hysteroscope into the cervical canal. At the end of the first day, we provided a hands-on session in training simulators. The practical part of the program is crucial in developing the trainees' technical skills and confidence in handling specialized instruments, particularly focusing on the use of the 30° fore-oblique hysteroscope. Trainees are divided into groups and assigned to one of three simulator stations, each outfitted with a dry lab simulator, monitor, light source, camera, and a 5 mm Bettocchi hysteroscope (Karl Storz, Tuttlingen, Germany). They become familiar with the fundamental components of hysteroscopic equipment, with a particular focus on assembling and disassembling the hysteroscope and understanding how it connects to the monitor, light source, and distension fluid pump (Figures 1A, 1B). Trainees also practice using hysteroscopic tools such as scissors and graspers, simulating basic procedures like endometrial polyp removal, uterine septum dissection, and adhesion management. Trainees who faced challenges during hands-on sessions were offered the opportunity to repeat procedures, time permitting, and were encouraged to attend future iterations of the course for reinforcement of skills.

**Table 1:** Topics covered.

Module topic	Subtopics covered
Indications & contraindications	Infertility, abnormal bleeding, recurrent miscarriage, uterine anomalies
Instrumentation & setup	Bettocchi hysteroscope, resectoscope, light source, camera, fluid pump
Operating theater preparation	Trolley layout, sterilization, draping protocol
Anesthesia & patient preparation	Local vs. general anesthesia, patient positioning, informed consent
Distension media & fluid deficit	Use of saline, monitoring fluid balance, prevention of overload
Optical system calibration	Use and orientation of 30° optics, scope alignment
Complication management	Uterine perforation, bleeding, fluid overload, vasovagal response
Patient counseling & consent	Expectations, risks, informed consent documentation
Pre- and post-operative care	Antibiotics, anti-adhesive gel, discharge protocols

**Figure 2:**
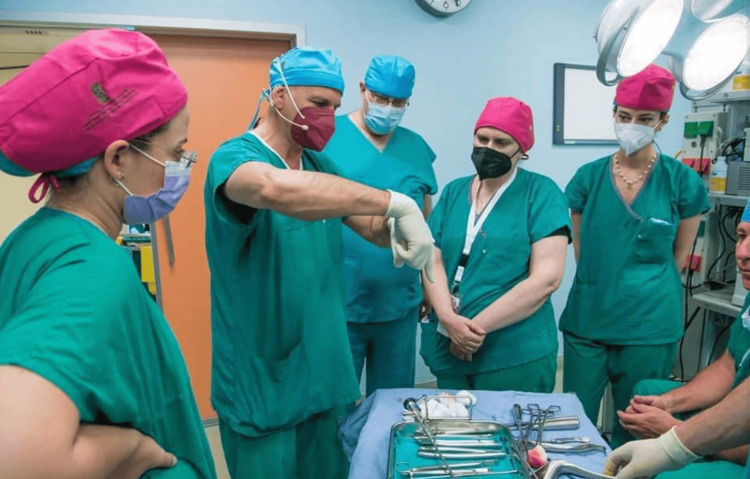
Trainees are getting familiar with the instrumentation, the Bettocchi hysteroscope, and the resectoscope.

On the second day, the practical training progresses to a more advanced level, with a smaller group of 10-15 trainees receiving one-on-one instruction in the operating room. Here, they practice using the Bettocchi hysteroscope and resectoscope under the guidance of an individual tutor (Figure 2 ). This session offers a more hands-on surgical experience, enabling trainees to perform a wide range of procedures, such as diagnostic hysteroscopy, removal of endometrial polyps or submucosal myomas (FIGO types 0-2), and the management of endometrial adhesions or uterine septum. Additionally, trainees are taught how to apply anti-adhesive gel and other preventive techniques to reduce the risk of intrauterine adhesion formation [19,20].

**Figure 3:**
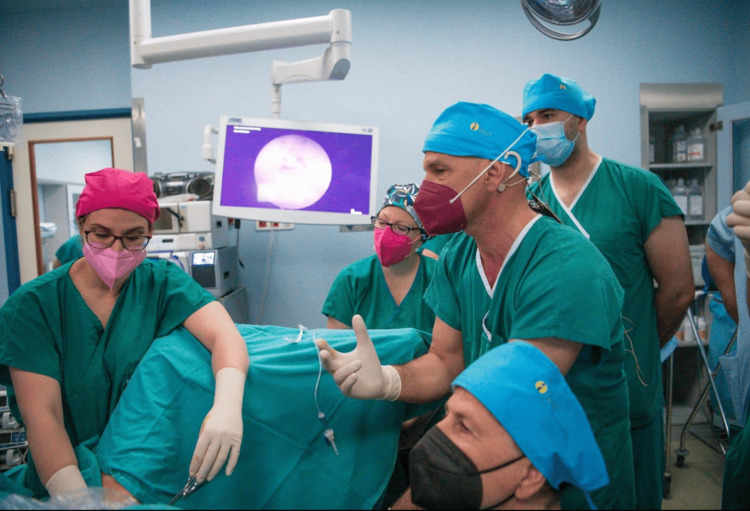
Diagnostic procedure demonstrated in real patients. Trainees are given the opportunity for hands-on training under the guidance of experienced tutors.

More than 20 patients were scheduled across four operating rooms to ensure that each trainee had the opportunity to perform at least two procedures under the supervision of experienced tutors. Tutors are encouraged to demonstrate a variety of techniques, highlighting the importance of surgical precision and prioritizing patient safety throughout the training (Figure 3). Live rebroadcasting of the operations is available for all participants to further discuss and analyze the cases. To minimize the risk of intraoperative complications, surgery time is typically kept under 30 minutes, while distention fluid loss is closely monitored. This controlled setting ensures patient safety during the procedure.

## Results

**Figure 4:**
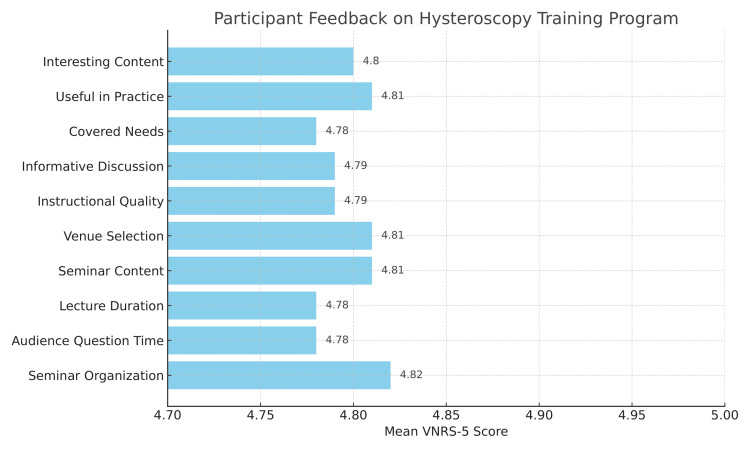
Seminar feedback. 1: very unsatisfied/not at all useful; 2: slightly satisfied/limited usefulness; 3: moderately satisfied/useful; 4: satisfied/very useful; 5: fully satisfied/extremely useful VNRS-5: 5-point verbal numeric rating scale

The hysteroscopy training program has been organized and continuously evaluated for seven consecutive years. Each year, all participants were requested to complete a self-administered questionnaire, composed of 10 items regarding the information obtained from the seminar. This questionnaire is designed to assess various aspects of the program, including content relevance, instructional quality, and practical application. We used a self-reported 5-point verbal numeric rating scale (VNRS-5) to evaluate our hysteroscopy course. Out of 245 participants who attended our course during seven years, 237 completed the questionnaire (Figure 4). Of the 237 respondents, 92% rated each session as either 4 or 5 on the VNRS-5 scale, suggesting a strong positive reception. Eight trainees (3.3%) did not submit the post-course questionnaire due to scheduling conflicts or early departure, and their responses were excluded from the analysis.

Given the above, it is clear that attendees found the content both engaging and highly relevant to their daily clinical practice, emphasizing the practical value of the topics covered. The hands-on sessions were rated as effective and beneficial, greatly contributing to the overall impact of the seminar.

Notably, the seminar’s organization, the smooth flow of sessions, and the time allocated for audience interaction were all praised by participants. The option of conference venue also contributed to a professional and welcoming atmosphere. Overall, the seminar was commended as a well-structured and valuable educational experience. According to the results of the questionnaire, the overall satisfaction of the participants was very high (4.8). The standard deviation of these ratings was 0.41 (suggesting that approximately 68% of responses fell between 4.39 and 5.0), and a statistical comparison against a neutral satisfaction level (3.0) yielded a p-value < 0.0001, confirming the significance of the observed high satisfaction.

The data collected through these surveys has provided valuable insights into the effectiveness of the program and areas for improvement. This ongoing evaluation process ensures that the training program is constantly refined and adapted to meet the evolving needs of practitioners. Feedback from participants has consistently been utilized to enhance both the curriculum and teaching methodologies, reinforcing the program's commitment to continuous improvement and high-quality training.

## Discussion

In this study, we described our experience from the training program developed by “Aretaieion” Hospital. It encompasses both theoretical, practical, and hands-on training, aiming to aid trainees in acquiring the expertise to autonomously perform diagnostic and operative hysteroscopy.

Most training alternatives utilize VR technology and dry or wet labs [21], few use training models, and even fewer include both theoretical and surgical components [22]. The theoretical part of our training program is vital for safe and effective hysteroscopic practice. It helps practitioners acquire the skills and insight needed to be able to make informed clinical decisions, ensuring precision and efficacy in their hysteroscopic procedures [23,24]. Simulation-based training and practice on models are also crucial, providing trainees the opportunity to familiarize themselves with the equipment and different procedures before performing them on live patients [25]. Nevertheless, unfortunately, several hospitals in Greece and Europe still cannot provide adequate training in hysteroscopy, and our live-patient training course is fundamental in hysteroscopy education, especially for young colleagues [26]. It exposes trainees to essential hands-on experience, crucial for developing technical skills and proficiency in handling hysteroscopic instruments within the uterine cavity. In addition, the learning process is further enhanced through mentorship by experienced tutors as well as through the opportunity to observe live procedures. This direct exposure to real clinical scenarios and techniques improves the trainees’ understanding and application of hysteroscopic skills [17].

Across the rest of Europe, hysteroscopy training courses are very diverse, yet they all share common training methodology [16-18,27-29]. They predominantly use video workshops, surgical models, and short clinical attachments that offer exposure to real-life scenarios. One of the most recognized and comprehensive training programs is presented by the European Society for Gynaecological Endoscopy (ESGE), the GET UP training course [30]. It is held annually, providing the opportunity for gynecologists at the beginning of their surgical career to develop the clinical and technical training required to perform minimally invasive procedures. Our program fully recognizes and shares the principles of the ESGE model program [29], such as psychomotor skills training in simulated environments, short interactive communication, panel-supported, on anatomical recognition, problem-solving and management, and prevention of complications [30]. On the other hand, we provide live surgery sessions under the guidance of experienced gynecologists, which is the major difference between the two courses.

Notably, Neveu et al. developed a curriculum that systematically adopts guidelines for hysteroscopy training based on expert consensus [18]. However, that approach is primarily theoretical, with practical training restricted to simulators and VR sessions. In an innovative approach, Chatzipapas et al. proposed a training model that incorporates practice on human uterine specimens obtained post-hysterectomy. This method provides a more realistic experience since human tissue is used; however, it lacks the crucial element of dealing with live patients, where hysteroscopic procedures are considerably more intricate and challenging [16]. Gambadauro et al. [17] presented a systematic report with recommendations on hysteroscopy training. Their evidence-based review indicated that routine training is largely theoretical, supplemented only by simulation courses. They raised concerns about whether this approach adequately prepares trainees for the complexity and unpredictability of real-life patients. Their findings suggest that simulator-based courses might not accurately predict clinical performance in actual patient settings [17]. Our training program provides invaluable one-on-one mentorship in live-surgery settings, offering immediate corrective feedback and reinforcing sound operative techniques through direct, personalized instruction. This program is especially beneficial for less experienced gynecologists, providing a safe and supportive environment to practice basic hysteroscope manipulations, while more experienced participants have the opportunity to improve their skills in more advanced operative procedures such as the management of intrauterine adhesions and removal of myomas FIGO type 2.

Hands-on training in live patients poses ethical, legal, and economic challenges [9]. These originate from the necessity to balance educational objectives with patient safety and rights, legal accountability, and the financial implications of such training. The primary ethical concern in hands-on training is patient safety and well-being. Prior to their participation, patients must be fully informed of the trainees’ role in their care and understand the possible implications. Detailed informed consent should be provided to all patients. From a legal perspective, involving trainees in medical procedures poses risks of malpractice and public liability. Errors or complications due to inexperience could provoke legal actions against the healthcare providers and institutions [9]. This renders strict supervision by experienced practitioners and rigorous training standards necessary. Furthermore, there is a need for clearly defined legal frameworks to offer guidance on the participation of trainees in patient care, ensuring that patient rights are protected and at the same time allowing trainees to gain the experience they need. We acknowledge that adaptations may be required in more litigious or ethically restrictive regions (e.g., Scandinavia or North America). These could include pre-operative risk stratification, trainee-specific malpractice coverage, or simulation-based alternatives. Moreover, from a financial point of view, the incorporation of hands-on training in live-patient settings can be associated with higher costs and total training duration. This can impact the efficiency of healthcare delivery in busy clinical settings.

Strengths and limitations

The limitation of the study is considered the low number of participants during the operating day of our course and the inability to perform more than two operations for each trainee. Subsequently, the post-course evaluation questionnaire was designed in-house and, while applied consistently, was not externally validated using psychometric analysis such as Cronbach’s alpha. This may influence the reliability of feedback measurement. Although satisfaction ratings were uniformly high, this may reflect self-selection bias, small tutor-to-trainee ratios, and the presence of constant supervision. Additionally, participants who encountered challenges often cited the supportive environment as a key strength, rather than expressing dissatisfaction. Despite high satisfaction scores suggesting program feasibility and acceptance, we recognize that these do not objectively assess surgical competency. Future studies should incorporate Objective Structured Assessment of Technical Skills (OSATS) scoring or complication tracking to evaluate skill acquisition. Finally, as program developers, we recognize the potential for bias in interpretation. This is mitigated by anonymized feedback collection, but future evaluations should involve third-party assessors.

The primary strength of our hysteroscopy training program is its focus on real patient-based learning rather than relying solely on theory or simulation. Trainees gain hands-on experience in live operative settings, working closely with experienced tutors throughout. This one-to-one supervision helps build both technical confidence and clinical judgment. By combining direct patient interaction with guided teaching, the program offers a more practical and in-depth understanding of hysteroscopic procedures, which many current training models often lack.

## Conclusions

Our curriculum stands out for its focus on hands-on operative experience and one-to-one tutoring. Unlike the predominantly theoretical and simulated approaches of other courses, our program immerses trainees in live-patient care, offering invaluable practical experience. This unique aspect of our training ensures that gynecologists not only learn the various techniques of hysteroscopy but also develop the skills and clinical judgment necessary to handle delicate and complex procedures in real patient settings. Our course structure is meticulously designed to connect theory and practice. Through working closely with experienced tutors and engaging directly with patients, trainees acquire a profound understanding of the different techniques applied in various hysteroscopic procedures.

In conclusion, the utilization of theoretical knowledge, practical skills, and real-life patient experience renders our course an innovation in hysteroscopy training, complementing the limitations of existing training models. Our commitment to continuous improvement, based on feedback and evolving medical practices, will ensure that the course remains at the forefront of hysteroscopy education.

## Figures and Tables

**Figure 5:**
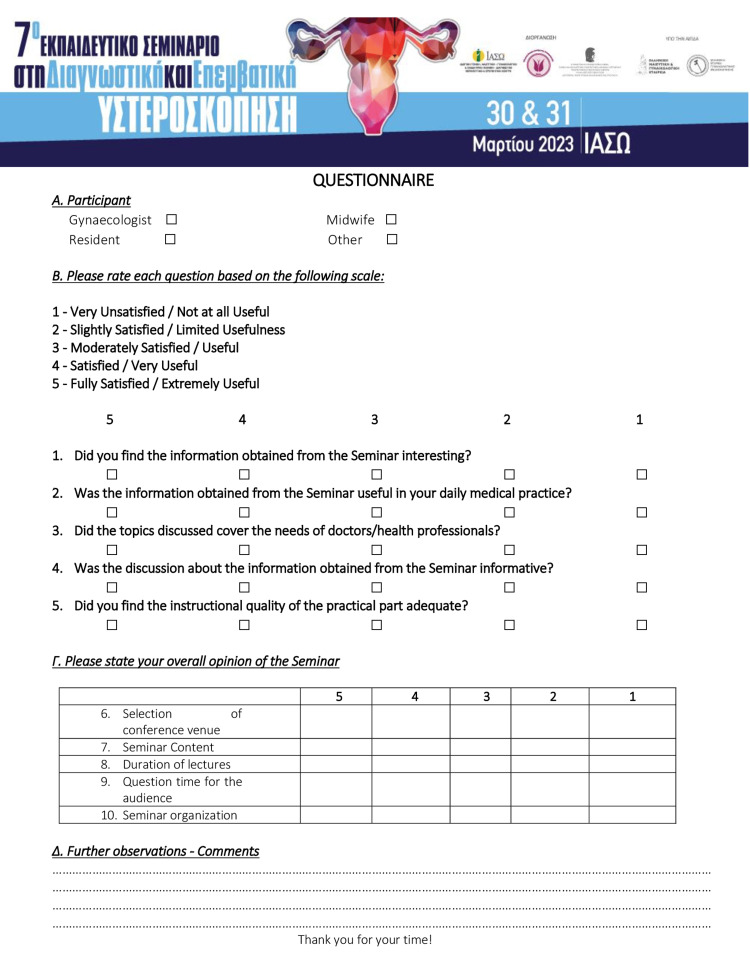
Evaluation questionnaire.
